# Supplementary material for: Splice-Junction-Based Mapping of Alternative Isoforms in the Human Proteome
Source: Cell Rep. Author manuscript; Available in PMC 2020 Jan 15. (PMC6961840; doi:10.1016/j.celrep.2019.11.026)
Supplement: 2 [file NIHMS1546469-supplement-2.zip › DF1/PXD010154/Lung/Lung_20_BBS1_EGQSAPLLSAHVNMPGSEGLAAPNRPLNPE.pdf]

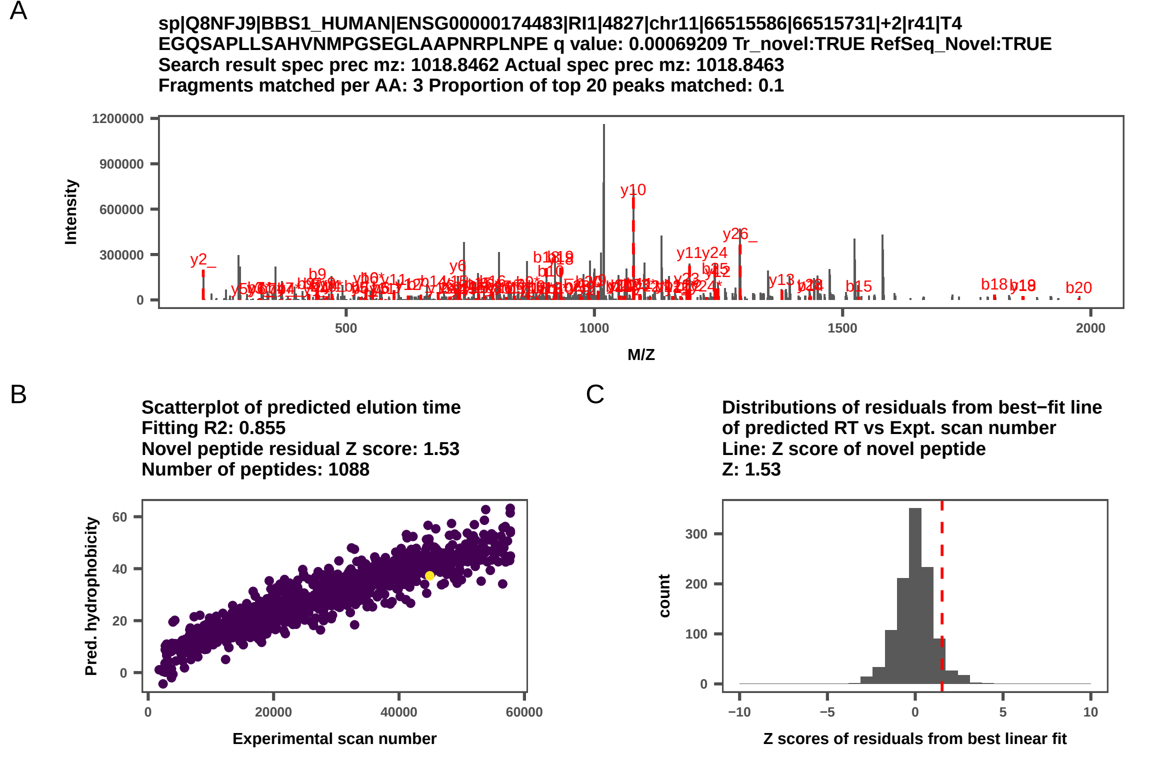
A
sp|Q8NFJ9|BBS1_HUMAN|ENSG00000174483|RI1|4827|chr11|66515586|66515731|+2|r41|T4
EGQSAPLLSAHVNMPGSEGLAAPNRPLNPE q value: 0.00069209 Tr_novel:TRUE RefSeq_Novel:TRUE
Search result spec prec mz: 1018.8462 Actual spec prec mz: 1018.8463
Fragments matched per AA: 3 Proportion of top 20 peaks matched: 0.1
Intensity
1200000
900000
600000
300000
0
y2_
y10
y26_
y11y24
y13
b15
b18
b20
M/Z
500
1000
1500
2000
B
Scatterplot of predicted elution time
Fitting R2: 0.855
Novel peptide residual Z score: 1.53
Number of peptides: 1088
Pred. hydrophobicity
60
40
20
0
0
20000
40000
60000
Experimental scan number
C
Distributions of residuals from best−fit line
of predicted RT vs Expt. scan number
Line: Z score of novel peptide
Z: 1.53
count
300
200
100
0
−10
−5
0
5
10
Z scores of residuals from best linear fit
